# Tocomin Restores Endothelium-Dependent Relaxation in the Diabetic Rat Aorta by Increasing NO Bioavailability and Improving the Expression of eNOS

**DOI:** 10.3389/fphys.2019.00186

**Published:** 2019-03-04

**Authors:** Saher F. Ali, Owen L. Woodman

**Affiliations:** School of Health and Biomedical Sciences, RMIT University, Bundoora, VIC, Australia

**Keywords:** eNOS, endothelium, diabetes, nitric oxide, oxidative stress, tocomin

## Abstract

We aimed to determine whether tocomin, an extract from palm oil that has a high tocotrienol content, was able to prevent diabetes-induced endothelial dysfunction. To induce type 1 diabetes streptozotocin (50 mg/kg) was injected into the tail vein of Wistar rats. Six weeks later the diabetic rats, and normal rats injected with citrate buffer, commenced treatment with tocomin (40 mg/kg/day sc) or its vehicle (peanut oil) for a further 4 weeks. Aortae isolated from diabetic rats had impaired acetylcholine (ACh)-induced endothelium-dependent relaxation compared to normal rat aortae but there was no change in endothelium-independent relaxation in response to sodium nitroprusside. By contrast, responses to ACh in aortae from diabetic rats treated with tocomin were not different to normal rats. In addition to impaired endothelium-dependent relaxation the diabetic aortae had increased expression of the NADPH oxidase Nox2 subunit, increased generation of superoxide and decreased expression of eNOS and all of these effects were prevented by tocomin treatment. Tocomin did not affect plasma glucose levels. The impaired response to ACh *in vitro* was maintained in the presence of TRAM-34 and apamin, selective inhibitors of calcium-activated potassium (K_*Ca*_) channels, indicating diabetes impaired the contribution of NO to endothelium-dependent relaxation. By contrast, neither diabetes nor tocomin treatment influenced EDH-type relaxation as, in the presence of L-NNA, an inhibitor of eNOS, and ODQ, to inhibit soluble guanylate cyclase, responses to ACh were similar in all treatment groups. Thus tocomin treatment improves NO mediated endothelium dependent relaxation in aortae from diabetic rats associated with a decrease in vascular oxidant stress but without affecting hyperglycaemia.

## Introduction

Diabetes is commonly associated with vascular dysfunction, particularly an impairment of endothelium-dependent relaxation, which is regarded as critical to the development of diabetes-induced vascular complications (De Vriese et al., 2000). Intensive control of blood glucose levels through the use of oral hypoglycaemic agents and insulin have been used to treat diabetes but they have proven to be ineffective in the prevention of the vascular complications of diabetes (Brown et al., 2010). In diabetes, hyperglycaemia-induced oxygen radical generation, mainly superoxide anion (O_2_^-^) radicals, play a key role in the pathogenesis of vascular complications (Giacco and Brownlee, 2010; Fiorentino et al., 2013; Hammes, 2018). This is mainly due to free radicals damaging the vascular endothelium which produces the potent vasorelaxant nitric oxide (NO) (Treuer and Gonzalez, 2015). Thus, there is a search for effective antioxidants that might prevent diabetes induced vascular dysfunction and its subsequent cardiovascular complications.

There has been considerable interest in the antioxidant activity and consequently in the potential use of vitamin E to reduce cardiovascular disease and its complications (Mathur et al., 2015; Schmidt et al., 2015). Vitamin E, mainly as α-tocopherol, has been reported to have beneficial effects on vascular function through its antioxidant activity, however, large-scale clinical trials (mainly using α-tocopherol) have produced disappointing results. There is emerging evidence that tocotrienols, which are structurally related to the tocopherols may possess superior antioxidant activity (Serbinova et al., 1991; Serbinova and Packer, 1994; Atkinson et al., 2008) and may also have distinct biological activities (Khanna et al., 2005; Sen et al., 2008; Aggarwal et al., 2010).

When investigating the capacity of tocotrienols to preserve endothelial function in the presence of oxidative stress *in vitro* we have previously shown that the individual isomers α-, δ-, and γ-tocotrienol failed to demonstrate any beneficial effect. However, tocomin, a mixture that contains a high proportion of tocotrienols with a small component of α-tocopherol, was able to reduce O_2_^-^ production and improve endothelium-dependent relaxation in rat aorta in the presence of chemically-induced oxidative stress (Ali and Woodman, 2015). Interestingly, a mixture of tocotrienols was able to mimic the protective effects of tocomin, but only in the additional presence of α-tocopherol. We have also previously demonstrated that 4-week treatment of obese rats with tocomin improves vascular function and reduces oxidative stress in the rat aorta by reducing the expression of the superoxide producing subunit of NADPH oxidase (Nox2) and improving the expression of the NO producing enzyme eNOS and its regulatory proteins Akt and CaM (Ali et al., 2016), both of which act to increase eNOS activity (Fleming, 2010). Therefore, in this study, we tested the hypothesis that treatment with the predominantly tocotrienol containing mixture tocomin would preserve endothelial function in aortae isolated from type 1 diabetic rats and we sought to determine the mechanism of any beneficial actions by investigating the effect of diabetes and tocomin on NO-mediated and endothelium dependent hyperpolarization (EDH)-type relaxation. We also determined the vascular expression of eNOS, proteins that positively (Akt, CaM) or negatively (caveolin-1) modulate eNOS activity and the NADPH oxidase subunit Nox2, an important contributor to vascular generation of O_2_^-^. This work was originally described in the Ph.D. thesis of SA (Ali, 2017).

## Materials and Methods

### Animals

All procedures were approved by the Animal Experimentation Ethics Committee of RMIT University (#1121) and conformed to the National Health and Medical Research Council of Australia code of practice for the care and use of animals for scientific purposes.

### Type 1 Diabetes

Male 6–8 weeks old Wistar rats (240–280 g) (Animal Resource Centre, Perth, WA, Australia) were randomly divided into two groups: normal (*n* = 20) and diabetic (*n* = 20). The rats were housed in groups of two under a light/dark cycle (12/12 h), in a temperature-controlled room (22°C), fed a standard chow diet (Specialty Feeds, Australia) and water was available *ad libitum*. Type 1 diabetes was induced by a single injection of streptozotocin (diabetic, STZ, 50 mg/kg) into the tail vein after an overnight fast. The control groups (normal) received an equivalent volume of vehicle (citrate buffer, 0.1 M) alone. Induction of diabetes was considered successful when blood glucose levels exceeded 15 mM (leading to exclusion of three rats from the no treatment group and two rats from the tocomin treatment group). Diabetic rats whose blood glucose concentration exceeded 25 mM or which had 5% or greater weight loss (compared to initial bodyweight) were administered with long-acting insulin (3–5 IU s.c. three injections per week. Protophane Novo Nordisk, Australia) to halt any further weight loss and promote health of the rat in the absence of euglycaemia. The rats were killed 10 weeks after STZ or vehicle injection by asphyxiation by CO_2_ inhalation, followed by decapitation, and their chests were then opened to isolate the thoracic aortae.

### Drug Administration

Tocomin was administered as we have previously described (Ali et al., 2016). Six weeks after induction of diabetes or injection of vehicle, tocomin treatment (40 mg/kg/day s.c.; normal *n* = 10, diabetic *n* = 10) or vehicle (peanut oil.; normal *n* = 10, diabetic *n* = 10) was commenced for a period of 4 weeks until the end of the experimental period. The final dose of tocomin was administered the day prior to euthanasia. We chose to deliver tocomin subcutaneously to ensure accuracy of dose delivery to each rat rather than by inclusion in the diet and therefore the oral route. Tocomin (Carotech, Malaysia) is a palm oil extract containing a tocotrienol-rich fraction (40%), palm olein (38%), and α-tocopherol (11%).

### Blood Glucose and Glycated Hemoglobin Levels

Blood glucose levels were measured weekly using blood collected from a needle prick to the tail vein and the blood glucose levels (BGL) were measured using a one-touch glucometer (Roche, Sydney, NSW, Australia). At the termination of the experiment blood samples were obtained from the carotid arteries after decapitation. Blood glucose was measured using the glucometer and glycated hemoglobin (HbA1c) was measured using an in2it^TM^ (II) analyser (Bio-Rad, Hercules, CA, United States).

### Assessment of Vascular Function

Vascular reactivity *in vitro* was assessed as we have previously described (Leo et al., 2011a,b; Ali et al., 2016). Following decapitation, the thoracic aorta was isolated and immediately placed in ice-cold Krebs bicarbonate solution (118 mM NaCl, 4.7 mM KCl, 1.18 mM MgSO_4_, 1.2 mM KH_2_PO_4_, 25 mM NaHCO_3_, 11.1 mM D-glucose, and 1.6 mM CaCl_2_). The aorta was then cleared of fat and connective tissue and cut into 2–3 mm long segments. Some additional segments of the thoracic aortae were used to measure superoxide production and protein expression. The aortic rings were mounted between two stainless steel wires, one of which was linked to an isometric force transducer (model FT03, Grass Medical Instruments, Quincy, MA, United States) connected to a Powerlab (AD Instrument Co., Sydney, NSW, Australia), and the other end anchored to a glass rod submerged in a standard 10 mL organ bath. The organ bath was filled with Krebs-bicarbonate solution. The bath medium was maintained at 37°C, pH 7.4 and continuously aerated with 95% O_2_, 5% CO_2_. Aortic rings were equilibrated for 45 min at a resting tension of 1 g, and then were pre-contracted with an isotonic, high potassium physiological salt solution (KPSS, 122.7 mM KCl, in which K^+^ ions replaced Na^+^ ions in the solution) for 20 min to achieve maximal contraction. After re-equilibration, the rings were sub-maximally contracted with phenylephrine (PE, 0.01–0.3 μM) and endothelial integrity was tested by addition of a single concentration of acetylcholine (ACh, 10^-5^ M). Where relaxation was greater than 80% (normal) or 50% (diabetic) of the pre-contraction the endothelium was considered to be intact and the aortic ring was included in the study.

Cumulative concentration response curves, firstly to ACh (0.1 nM–10 mM) and subsequently to sodium nitroprusside (SNP, 0.1 nM–10 mM), were determined using aortic rings contracted with phenylephrine (10^-8^ to 10^-7^ M) to 40–60% of maximal contraction. Responses to ACh and SNP were also tested in the presence or absence of inhibitors of small calcium activated potassium channels (SK_*Ca*_, apamin, 1 μM) intermediate calcium -activated potassium channels (IK_*Ca*_, TRAM-34, 1 μM), nitric oxide synthase (NOS), N-nitro-l-arginine methyl ester (L-NAME, 100 μM), and soluble guanylate cyclase (sGC), 1H-[1,2,4] oxadiazolo[4,3-a]quinoxalin-1-one (ODQ, 10 μM) to investigate the role of NO and endothelium-dependent hyperpolarization (EDH)-type relaxation through the opening of potassium channels in the relaxant responses. All treatments were added to the baths 20 min prior to conducting the concentration–response curves. After the initial concentration-response curves to ACh the aortic rings were washed with physiological saline, allowed to regain basal tension and exposed to the same inhibitors before contraction to PE and responses to SNP were determined. The negative logarithm of the concentration at which 50% relaxation occurred (pEC_50_) and maximum relaxation (R_*max*_) values were calculated from the individual cumulative dose response curves using Graphpad Prism 6.

### Superoxide Production in Aorta

Superoxide production in aortic rings was measured using lucigenin-enhanced chemi-luminescence using procedures we have described previously (Ali et al., 2016). The thoracic aorta was isolated, cleared of fat and connective tissue, and cut into 2–3 mm long segments which Krebs-HEPES buffer. Aortic ring segments were incubated at 37°C for 45 min in Krebs-HEPES buffer in a Costar cell culture plate in the presence of NADPH (100 mM) as a substrate for NADPH oxidase and DETCA (3 mM), to inactivate endogenous SOD and therefore maximize superoxide detection. In addition, superoxide was measured in the presence of diphenylene iodonium (DPI, 5 mM), a flavoprotein inhibitor that inhibits NADPH oxidase, as a positive control. 300 μL of Krebs-HEPES buffer containing lucigenin (5 mM) were placed into a 96-well Optiplate, and superoxide production was measured. The aortic segments were then transferred to the corresponding wells of the optiplate and the photon emission was recounted. Tissue was dried in a 37°C oven and weighed. Superoxide production was quantified as arbitrary units of superoxide production and expressed as a ratio to dry tissue mass (AU/mg dry tissue).

### Protein Expression

To measure vascular expression of selected proteins Western blots were performed as described previously (Miller et al., 2005; Leo et al., 2011b; Ali et al., 2016) with some modifications. Briefly, the tissues were homogenized in 200 mL of ice-cold lysis buffer [100 mM NaCl, 10 mM Tris, 2 mM EDTA, 0.5% w/v sodium deoxycholate, 1% vol/vol Triton X-100, pH 7.4, protease, and phosphatase inhibitor cocktails (Roche, Sydney, NSW, Australia)], and the total protein concentration of the samples was quantified using the Bio-Rad Bradford assay. Protein samples were thawed and heated at 95°C for 5 min. Equal amounts of protein homogenate (30 μg) were loaded onto the SDS-PAGE gels and run at 100 V until the lowest molecular weight marker was at the bottom of the gel. Proteins in the SDS gel were transferred onto a nitrocellulose membrane (0.45 μm pore size) using a wet transfer at 90 V for 90 min at 4°C. Membranes were blocked in 0.25% BSA in tris-base saline Tween 20 (TBST pH 7.4) for 1 h at room temperature and incubated with anti-mouse/rabbit primary antibodies probing for proteins of interest (eNOS, Nox2, and caveolin-1, BD Transduction Laboratories, Lexington, KY, United States; Akt and pAkt, Cell Signaling, Danvers, MA, United States; calmodulin, Merck, Millipore, Australia; all antibody dilutions were 1:1000 in TBST, overnight, 4°C). Membranes were washed the following the day (3 ×_10 min) in TBST followed by incubation in HRP-linked mouse/rabbit secondary antibody for 1 h at room temperature. Membranes were washed and then developed using a ChemiDoc XRS (Bio-Rad, Sydney, NSW, Australia). All proteins were detected using either enhanced chemiluminescence (Amersham, GE Healthcare, Sydney, NSW, Australia) or Supersignal West Femto (Thermo Scientific, Rockford, IL, United States). Membranes were then blocked in 0.1% sodium azide (HRP inhibitor) for 1 h at room temperature and then re-probed with the loading control primary antibody [anti-mouse/rabbit β-actin (1:2000)] as described above.

### Reagents

All drugs were purchased from Sigma Aldrich except for ACh perchlorate (BDH Chemicals, Poole, Dorset, United Kingdom) and tocomin (Carotech, Malaysia). All drugs were dissolved in distilled water, with the exception of tocomin that was dissolved in peanut oil. All antibodies were sourced from Genesearch Australia or Merck Millipore (United States) and were diluted to 1:1000 in TBST pH 7.4 except for β-actin which was diluted to 1:5000.

### Statistical Analyses

All results are expressed as mean ± SEM, where *n* represents the number of animals per group. Concentration-response curves from the rat-isolated aortae were constructed and fitted to a sigmoidal curve using non-linear regression (Graphpad Prism version 6.0, Graphpad Software, San Diego, CA, United States) to calculate the sensitivity of each agonist (pEC_50_). Maximum relaxation (R_*max*_) was measured as a percentage of the pre-contraction to phenylephrine. Group pEC_50_ and R_*max*_ values were compared using a two-way ANOVA with *post hoc* analysis using Sidak’s test (vascular function) or Dunnet’s test (superoxide production and protein expression) as appropriate. *p* < 0.05 was considered statistically significant.

Superoxide levels from rat aortic rings are expressed as arbitrary units ± SEM normalized to dry tissue weight. Results were compared by one-way ANOVA with a *post hoc* Dunnett’s test. *p* < 0.05 was considered statistically significant.

All western blotting results were quantified by densitometry using ImageLab software (Bio-Rad, Sydney, NSW, Australia) and expressed as a densometric ratio of the primary protein to β-actin ± SEM. The quantification for the expression of pAkt is expressed as a ratio of pAkt to Akt ± SEM.

## Results

### The Effect of Diabetes and Tocomin Treatment on Body Weights, Blood Glucose and HbA1c

The body weight of normal rats was significantly greater than that of diabetic rats (Table 1) at the end of the experimental period. The blood glucose and HbA1c levels of diabetic rats was significantly greater than that of the normal rats (Table 1). 4-week treatment had no effect on bodyweight, blood glucose or HbA1c levels (Table 1).

**Table 1:** Mean body weight, fasting blood glucose, and HbA1c levels for normal and diabetic treatment groups.

	*n*	normal	*n*	diabetic	*n*	Normal + tocomin	*n*	Diabetic + tocomin
**Body Weight (g)**								
Initial (*t* = 0 weeks)	9	250 ± 8	7	262 ± 6	7	265 ± 9	7	255 ± 7
*t* = 6 weeks	9	336 ± 8	7	323 ± 15	7	341 ± 6	7	318 ± 13
Final (*t* = 10 weeks)	9	522 ± 17	7	381 ± 22*	7	552 ± 17	7	374 ± 30*
Blood Glucose (mM)	9	6.8 ± 0.3	7	32.2 ± 1.2*	8	7.4 ± 0.7	7	29.3 ± 3.1*
HbA1c (%)	9	5.7 ± 0.2	7	13.3 ± 0.3*	7	5.5 ± 0.2	7	11.8 ± 1.3*

^∗^*Significantly different to normal. Results are shown as mean ± SEM. Two-way ANOVA, Dunnet’s test, *p* < 0.05*.

### The Effect of Diabetes and Tocomin Treatment on Superoxide Production in the Rat Aorta

**Figure 1:**
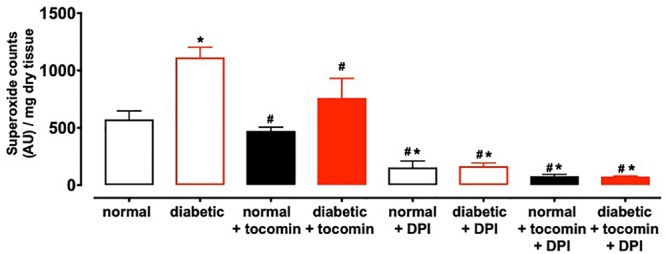
Superoxide generated in rat aorta in the presence of NADPH from normal, diabetic, and tocomin treated (normal + tocomin/diabetic + tocomin) and in the presence of DPI. Data is expressed as mean ± SEM. ^∗^Significantly different to normal. ^#^Significantly different to diabetic. Results are shown as mean ± SEM. *P* < 0.05. Two-way ANOVA, Dunnett’s multiple comparisons test. *n* = 3–6 experiments.

Nox2- induced superoxide production was significantly elevated in diabetic rat aortae in comparison to the normal rat aorta. Treatment of the diabetic rats with tocomin (40 mg/kg/day) significantly attenuated superoxide production in the aortae from diabetic rats. The presence of DPI, a non-selective inhibitor of NADPH oxidase, in the assay *in vitro* decreased superoxide levels detected in the aorta from normal and diabetic rats (Figure 1).

### Effect of Diabetes and Tocomin Treatment on Endothelial Function

**Figure 2:**
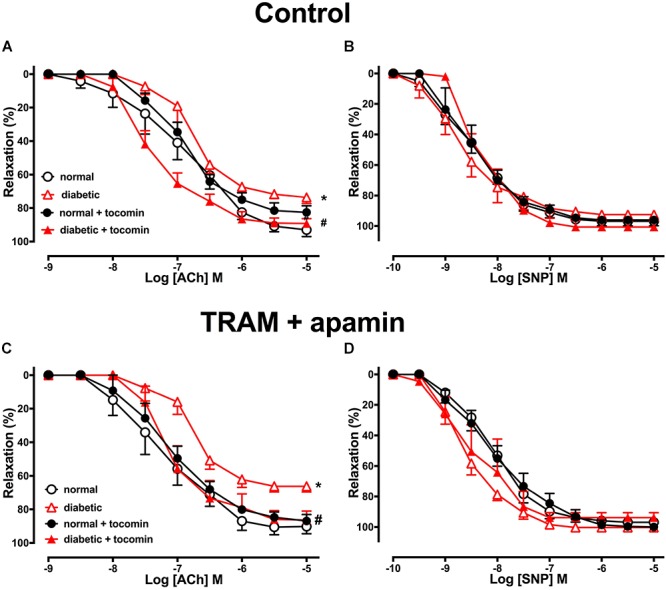
Cumulative concentration–response curves to ACh and SNP in endothelium-intact aortae isolated from normal, diabetic, and tocomin treated (normal + tocomin/diabetic + tocomin) rats in control **(A,B)** and in the presence of Tram + apamin **(C,D)**. ^∗^R_*max*_ Significantly different to normal. ^#^R_*max*_ Significantly different to diabetic. *P* < 0.05. Data is expressed as mean ± SEM. Sidak’s multiple comparison test. *n* = 3–7. See Table 2 for values and statistical comparison.

The maximum response (R_*max*_), but not the sensitivity (pEC_50_), to the endothelium-dependent relaxant ACh was significantly reduced in the aortae from diabetic compared to normal rats (Table 2 and Figure 2A). Responses to SNP were not affected by diabetes (Table 2 and Figure 2B). Treatment of the diabetic rats with tocomin (40 mg/kg/day sc) for 4 weeks significantly increased the maximum response to ACh in the aorta from diabetic, but not the normal, rats (Table 2 and Figure 2A). Tocomin treatment did not affect the endothelium-independent relaxation to SNP in any group (Table 2 and Figure 2B).

**Table 2:** The effect of 4-week tocomin treatment on ACh-induced endothelium-dependent and SNP-induced endothelium-independent relaxation of rat aortae during diabetes.

		ACh			SNP	
	*n*	pEC_50_ (M)	R_*max*_ (%)	*n*	pEC_50_ (M)	R_*max*_ (%)
***Normal (vehicle)***						
Control	7	6.95 ± 0.23	93 ± 4	6	8.42 ± 0.15	97 ± 3
Tram + apamin	5	7.27 ± 0.21	91 ± 5	5	8.38 ± 0.40	97 ± 1
L-NNA	3	6.51 ± 0.26*	16 ± 3*	3	8.21 ± 0.27	92 ± 5
L-NNA + ODQ	8	6.51 ± 0.26*	8 ± 2*	8	6.00 ± 0.30*	28 ± 8*
***Diabetic (vehicle)***						
Control	7	6.75 ± 0.13	76 ± 4$	7	8.54 ± 0.17	93 ± 3
Tram + apamin	5	6.67 ± 0.06	72 ± 4$	5	8.80 ± 0.29	100 ± 2
L-NNA	3	6.73 ± 0.21	28 ± 4*$	3	8.71 ± 0.16	103 ± 2
L-NNA + ODQ	5	6.73 ± 0.29	10 ± 4*	3	6.58 ± 0.24*	48 ± 5*
***Normal + tocomin (40 mg/kg/day)***						
Control	7	6.93 ± 0.09	83 ± 4	6	8.36 ± 0.25	96 ± 2
Tram + apamin	3	6.79 ± 0.10	82 ± 5	3	8.10 ± 0.20	100 ± 2
L-NNA	6	6.88 ± 0.13	23 ± 8*	6	8.50 ± 0.26	96 ± 5
L-NNA + ODQ	7	6.90 ± 0.06	11 ± 7*	5	5.95 ± 0.20*	48 ± 8*
***Diabetic + tocomin (40 mg/kg/day)***						
Control	7	7.38 ± 0.16	89 ± 3#	6	8.54 ± 0.14	101 ± 4
Tram + apamin	5	7.1 ± 0.10	80 ± 5	4	8.56 ± 0.33	94 ± 3
L-NNA	6	6.68 ± 0.16	38 ± 5*	6	8.47 ± 0.23	100 ± 3
L-NNA + ODQ	7	6.80 ± 0.21	16 ± 7*	6	6.26 ± 0.48*	48 ± 8*

^∗^*Significantly different compared to control in the same treatment group. ^$^Significantly different compared to the same treatment in the normal group. ^#^Significantly different to same treatment in the diabetic group. Results are shown as mean ± SEM. Two-way ANOVA, *p* < 0.05. Sidaks multiple comparison test*.

*Table illustrates the pEC_50_ and R_*max*_ values for ACh in aortic rings from normal, diabetic, and tocomin treated (normal + tocomin/diabetic + tocomin) rats. The effect of various chemical inhibitors is shown. *n* = no. of experiments*.

In the presence of inhibitors of SK_*Ca*_ (apamin) and IK_*Ca*_ (TRAM-34), ACh-induced relaxation is predominantly mediated by NO. Under those conditions the maximum response to ACh was significantly decreased in the diabetic rat aorta compared to the normal aorta (Table 2 and Figure 2C). Following 4-week treatment with tocomin, there was no change in ACh-sensitivity in aortae from the normal rats, but the maximum response to ACh was significantly increased in the diabetic rat aorta by the tocomin treatment. This suggests that tocomin treatment improves NO mediated relaxation. The presence of TRAM-34 and apamin had no effect on the endothelium-independent relaxant responses to SNP in any group (Table 2 and Figure 2D).

**Figure 3:**
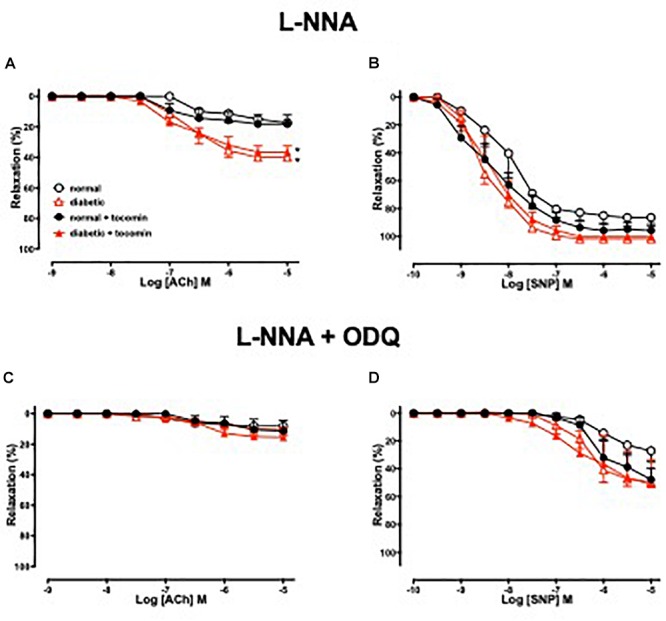
Cumulative concentration–response curves to ACh and SNP in endothelium-intact aortae isolated from normal, diabetic, and tocomin treated (normal + tocomin/diabetic + tocomin) rats in the presence of L-NNA **(A,B)** and L-NNA + ODQ **(C,D)**. Data is expressed as mean ± SEM. ^∗^*p* < 0.05. Sidak’s multiple comparison test. *n* = 4–8. See Table 2 for values and statistical comparison.

Figure 3 illustrates the responses to ACh and SNP in the presence of L-NNA with or without the additional presence of ODQ. L-NNA alone reduced the maximum relaxation to ACh in the aortae of normal and diabetic rats but importantly when comparing the maximum relaxation in the presence of L-NNA the responses are greater in the diabetic rats compared to normal. Further, in the presence of L-NNA, tocomin treatment had no effect on the response to ACh in normal or diabetic rat aortae (Table 2 and Figure 3A). In the presence of the sGC inhibitor ODQ in addition to L-NNA, ACh-induced relaxation was not different in the aortae from diabetic rats compared to normal rats (Table 2 and Figure 3C), and tocomin did not affect responses in either group.

L-NNA did not significantly affect responses to SNP (Figure 3B) but, the addition of ODQ, significantly reduced the sensitivity and maximum response to SNP in aortae from both normal and diabetic rats (Table 2 and Figure 3D). Tocomin had no effect on responses to SNP under any condition.

### The Effect of Diabetes and Tocomin Treatment on Nox2, eNOS, and Modulatory Proteins

**Figure 4:**
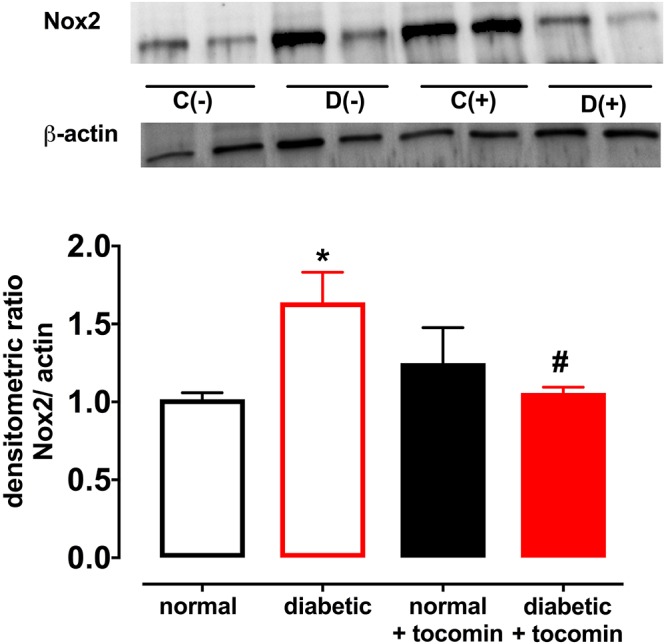
Protein expression of NADPH oxidase (Nox2) from isolated aortae from normal, diabetic, and tocomin treated (normal + tocomin/diabetic + tocomin) rats. Representative blots are shown for each corresponding graph. ^∗^Significantly different to normal. ^#^Significantly different to diabetic. Results are shown as means ± SEM. *P* < 0.05. Two-way ANOVA Dunnett’s multiple comparison test. *n* = 3–5 experiments.

**Figure 5:**
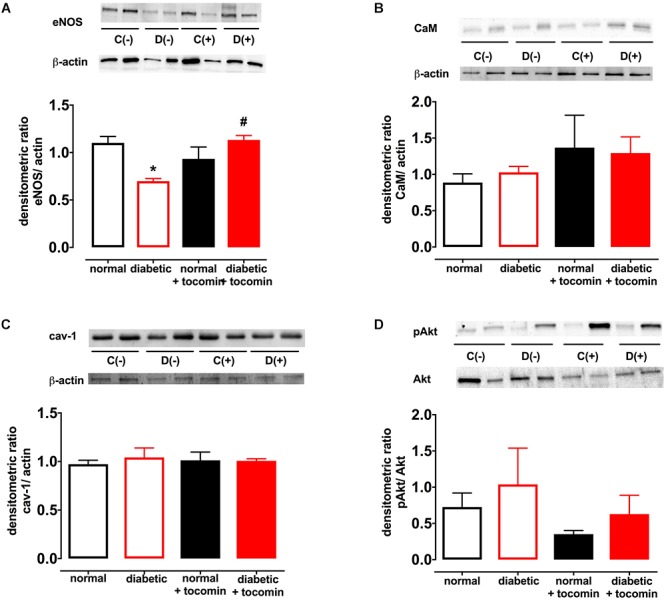
Protein expression of total eNOS **(A)**, calmodulin-1 **(B)**, caveolin-1 **(C)**, and pAkt/Akt **(D)** from isolated aortae from normal, diabetic, and tocomin treated (normal + tocomin/diabetic + tocomin) rats. Representative blots are shown for each corresponding graph. ^∗^Significantly different to normal. ^#^Significantly different to diabetic. Results are shown as means ± SEM; *p* < 0.05. Two-way ANOVA Dunnett’s multiple comparisons test. *n* = 3–5 experiments.

Diabetes significantly increased the expression of Nox2 in aortae from diabetic in comparison to the normal rats (Figure 4). The total expression of the NO producing enzyme eNOS was significantly lower in aortae from diabetic in comparison to normal rats (Figure 5A). Expression of either calmodulin (CaM) or caveolin-1 was not affected by diabetes. The proportion of Akt that was phosphorylated was also not different in the diabetic rats in comparison to the normal rats (Figure 5B–D). Treatment with tocomin reversed the diabetes-induced changes in eNOS and Nox2 expression (Figure 4, 5A).

This study has demonstrated that treatment of type 1 diabetic rats with tocomin, a palm oil extract rich in tocotrienols (40%) and to a lesser extent α-tocopherol (11%), reduces the levels of oxidative stress and selectively improves endothelium-dependent relaxation in aortae. As has been previously reported, NO is the major contributor to endothelium-dependent relaxation in the rat aorta (Zhao et al., 2015) and type 1 diabetes significantly impaired the contribution of NO to ACh-induced relaxation whereas the contribution EDH type relaxation was slightly increased. Tocomin treatment significantly increased the contribution of NO to relaxation in diabetic aortae without affecting EDH type relaxation. The improved NO responses were accompanied by a decrease in vascular superoxide production, a decreased expression of Nox2 and increased expression of eNOS. Thus, tocomin may both increase NO synthesis and improve NO bioavailability by decreasing its inactivation by superoxide.

## Discussion

### Effect of Diabetes and Tocomin on Endothelial Function

Responses to the endothelium-dependent relaxant ACh were selectively impaired in aortae from diabetic rats. There was a significantly greater level of O_2_^-^ generation in arteries from diabetic rats, in comparison to normal rat aortae, which was accompanied by an increase in Nox2 expression as we and others have previously described (Hink et al., 2001; Leo et al., 2011b). The increase in O_2_^-^ generation was accompanied by an impairment of endothelium-dependent relaxation to ACh, whereas the endothelium-independent dilator SNP caused similar responses in normal and diabetic vessels. The impaired relaxation was likely due to a decreased production and/or increased inactivation of endothelium-derived NO (Higashi et al., 2009) rather than any change in smooth muscle function given the maintenance of the responses to the NO donor SNP. Four-week tocomin treatment was able to significantly attenuate O_2_^-^ generation and reduce Nox2 expression in the diabetic rat aortae. This may be at least in part due to the capacity of tocomin, when administered to the rats, to acutely scavenge O_2_^-^ *in vivo* as we have previously reported to occur *in vitro* (Ali and Woodman, 2015). A similar study conducted by Keegan and colleagues has demonstrated that feeding type 1 diabetic rats 1% α-tocopherol for 2 months improved endothelial function in the aorta (Keegan et al., 1995). Although the mechanism of improvement was not investigated, the improvement in endothelial function was attributed to the antioxidant activity of α-tocopherol that reduced oxidative stress in the aorta and improved NO-mediated relaxation. The results from this study are also consistent with our previous study that demonstrated tocomin treatment reduced O_2_^-^ production in aortae from obese rats in the presence of oxidative stress by reducing Nox2 expression (Ali et al., 2016).

The decrease in Nox2 expression in the diabetic rat aortae observed in this study may be due to decreased protein kinase C (PKC) activation. PKC is a known positive regulator of Nox2 (Seshiah et al., 2002). A study conducted by Clement et al. (Clement et al., 1997) demonstrated that the treatment of rat aortic smooth muscle cells with *α*-tocopherol inhibited PKC activation. This α-tocopherol-induced inhibition of PKC was believed to be due to α-tocopherol acting at a cellular level in addition to its antioxidant activity (Carew et al., 1987; Esterbauer et al., 1992). We hypothesize that the tocomin-induced decrease in Nox2 expression that has been observed in this study could be explained by a similar mechanism, i.e., tocomin that is rich in tocotrienols, inhibits lipid peroxidation through its antioxidant function. Inhibition of lipid peroxidation leads to decrease activity of the diacylglycerol (DAG) kinase enzyme that is a stimulator of PKC. By reducing DAG kinase dependent PKC activation, tocomin may decrease PKC activation and subsequent Nox2 expression. A study by Lee et al. (1996) demonstrated that vitamin E (α-tocopherol) prevented hyperglycemia-induced activation of the DAG-PKC pathway in vascular smooth muscle cells by decreasing DAG kinase activity. As tocomin contains tocotrienols that share structural similarity and functionality to α-tocopherol, in addition to α-tocopherol itself, it may be acting through a similar mechanism.

### The Effect of Diabetes and Tocomin Treatment on NO-Mediated Relaxation and the Expression of eNOS and Its Regulatory Proteins

To investigate the influence of diabetes and tocomin treatment on the NO-mediated component of endothelium-dependent relaxation we examined responses to ACh in the presence of TRAM-34 plus apamin to eliminate the EDH-type component of relaxation. In the presence of the SK_*Ca*_ and IK_*Ca*_ channel blockers, the ACh-induced relaxation was significantly impaired in aortae from the diabetic rats compared to normal rats demonstrating that diabetes impaired NO-mediated relaxation. The NO mediated relaxation was significantly improved in the aortae from tocomin treated diabetic rats indicating that increased NO activity contributed to the tocomin-induced improvement in endothelium-dependent relaxation. A limitation of this study was that we did not investigate a potential role for prostanoids in the observed endothelium-dependent responses. It has been reported that diabetes may increase prostanoid-mediated endothelium-dependent contraction which would exacerbate endothelial dysfunction (for review Vanhoutte et al., 2009). It is notable, however, that in this study inhibition of eNOS with L-NNA eliminated any difference in the responses to ACh between the tocomin treated and untreated diabetic aortic rings suggesting that enhanced NO release could account for the beneficial effects of tocomin.

The impaired NO-mediated relaxation was accompanied by a decreased expression of eNOS. This may have been due to increased superoxide production resulting from increased Nox2 expression. Increased free radical activity in the vasculature has been demonstrated to lead to a decreased bioavailability of NO due to O_2_^-^ reacting with NO and forming the cytotoxic molecule peroxynitrite (ONOO^-^) (Heitzer et al., 2000). Further, tetrahydrobiopterin (BH_4_), a crucial cofactor of eNOS, is sensitive to oxidation by ONOO^-^ (Cosentino and Luscher, 1998). Where there is elevated oxidative stress, such as in diabetes, oxidation of BH_4_ can decrease eNOS activity causing uncoupling of the enzyme resulting in further generation of O_2_^-^ (Kuzkaya et al., 2003). Treatment of the diabetic rat aortae with tocomin improved eNOS expression in this study. This could be due to the antioxidant effect of tocomin to decrease uncoupling of eNOS in the diabetic aortae. The potential for an antioxidant to decrease eNOS uncoupling has been demonstrated previously where the treatment of diabetic rats with 3′,4′-dihydroxyflavonol decreased the uncoupling of eNOS in the mesenteric vasculature (Leo et al., 2011a). The antioxidant activity of tocomin, as evidenced by a decrease in vascular O_2_^-^ production, is also likely to have increased NO bioavailability leading to improved endothelium-dependent relaxation. The level of eNOS activity may be influenced by the expression of regulatory proteins either positively (e.g., Akt, CaM) or negatively (e.g., caveolin-1) (Fleming, 2010), however, in this study the expression of these proteins was not affected by diabetes or tocomin treatment nor was there any effect on Akt phosphorylation.

An additional beneficial action of tocomin in this study to reduce vascular oxidative stress was to decrease expression of the vascular NADPH oxidase subunit Nox2 in the diabetic rat aortae. The observation that DPI *in vitro* decreased O_2_^-^ is consistent with NADPH oxidase acting as the main source of the vascular oxidative stress. Increased Nox2 expression is known to impact vascular function. This was demonstrated by Violi et al. (2009) where subjects with a genetic Nox2 deficiency had enhanced endothelium-dependent relaxation. The ability of antioxidants to decrease Nox2 expression has also been previously reported. A study conducted by Wang et al. (2016) demonstrated that the antioxidant geraniol improved endothelium-dependent relaxation of the aortae of mice fed a high fat diet by down regulating Nox2 expression. Another study reported diabetes impaired endothelial function in the mesenteric arterioles of type 1 diabetic mice which was restored with the treatment with the antioxidant 3′,4′-dihydroxyflavonol (Leo et al., 2011a). Thus, it can be suggested that the decrease in Nox2 expression as observed in this study may be due to tocomin scavenging O_2_^-^ in the diabetic aortae and reducing oxidative stress in the vasculature.

### The Effect of Diabetes and Tocomin Treatment on EDH-Type Relaxation

To investigate how diabetes affects EDH-type relaxation and whether tocomin improves endothelium-dependent relaxation through an improvement in EDH-type relaxation we investigated endothelium-dependent relaxation in the presence of the eNOS inhibitor L-NNA alone and with ODQ. L-NNA alone significantly reduced endothelium-dependent relaxation in both normal and diabetic rat aortae, interestingly however, the maximum response to ACh was significantly larger (Figure 3A) in the diabetic in comparison to the normal rat aortae. This indicates that there may be an enhanced EDH contribution to relaxation in diabetes in an unsuccessful attempt to preserve endothelium-dependent relaxation when the contribution of NO is impaired. This is consistent with our previous observations (Joshi and Woodman, 2012) that EDH makes a greater contribution to ACh-induced relaxation of aortae from type 1 diabetic rats. Although we did not test a possible role for prostanoids in the current study we previously found that the cyclooxygenase inhibitor indomethacin had no effect on endothelium-dependent relaxation of aortae from normal or diabetic rats (Joshi and Woodman, 2012). There was no significant difference between responses to ACh in the presence of L-NNA alone and those in the presence of L-NNA plus ODQ within any group suggesting that there was no role for NO derived from any source other than through NOS. It was notable that tocomin had no significant effect on responses to ACh in the presence of L-NNA plus ODQ in either normal or diabetic arteries indicating that treatment did not affect EDH-type relaxation.

## Conclusion

There are many animal and human studies that demonstrate the link between ROS and the development of CVD (Ritchie et al., 2017) including in diseases associated with diabetes and obesity (Roberts et al., 2005; Stump et al., 2005). There has also been extensive research on the effect of antioxidants including vitamin E (predominantly α-tocopherol) in the prevention and treatment of cardiovascular disease, but despite many positive results in animal studies, they have not consistently translated to positive outcomes in clinical trials (Leopold, 2015). There is also growing evidence that tocotrienols possess other actions in addition to their potent antioxidant activity (Serbinova et al., 1991) such as cholesterol lowering (Pearce et al., 1992) and antithrombotic properties (Baliarsingh et al., 2005). Further, we have previously demonstrated (Ali and Woodman, 2015) that the combination of tocotrienols with α-tocopherol is more effective at preserving endothelial function in the presence of oxidative stress than either α-tocopherol or tocotrienols alone. We have also demonstrated that treatment of rats fed a high-fat western diet with tocomin attenuates the diet-induced vascular dysfunction by improving NO bioavailability and increasing the expression of eNOS (Ali et al., 2016).

This study demonstrated that the *in vivo* treatment of diabetic rats with the tocotrienol-rich extract of palm oil; tocomin, increased NO activity to improve endothelium-dependent relaxation. Tocomin did not affect diabetes induced hyperglycaemia or weight-loss but did attenuate the vascular oxidative stress in the aortae. Tocomin also reduced O_2_^-^ production in the diabetic aortae which was directly correlated with a decrease in Nox2 expression.

A further positive outcome of tocomin treatment in the diabetic rats was increased expression of eNOS in the aortae. This could be due to the antioxidant function of tocomin that decreases the oxidation of eNOS cofactors such as BH_4_, therefore preventing uncoupling of eNOS. The beneficial actions of tocomin observed in these diet-induced model of diabetes suggest that it may have potential to be used as a therapeutic agent to prevent vascular disease in diabetes.

## Data Availability

All datasets generated for this study are included in the manuscript and/or the supplementary files.

## Author Contributions

SA and OW contributed to the study design. SA performed the experiments, statistical analysis, and wrote the first draft of the manuscript. Both authors contributed to manuscript revision, read, and approved the submitted version.

## Conflict of Interest Statement

The authors declare that the research was conducted in the absence of any commercial or financial relationships that could be construed as a potential conflict of interest.
